# Trials in advanced Hodgkin's disease: asking the right questions at the right time

**DOI:** 10.1054/bjoc.2001.1840

**Published:** 2001-05

**Authors:** J Sweetenham

**Affiliations:** Division of Medical Oncology-F704, University of Colorado Health Sciences Center, 4200 East Ninth Avenue, Denver, CO, 80262, USA


*British Journal of Cancer* (2001) **84**(10), 1291–1292
© 2001 Cancer Research Campaign
doi: 10.1054/ bjoc.2001.1840, available online at http://www.idealibrary.com on IDEAL®

http://www.bjcancer.com

Editorial

### Trials in advanced Hodgkin's disease: asking the right questions at the right time

**J Sweetenham**

University of Colorado Health Sciences Center, Division of Medical Oncology-F704, 4200 East Ninth Avenue, Denver CO 80262, USA

The treatment of advanced Hodgkin's disease poses unique challenges for those involved in the design of randomized clinical trials. The disease predominantly affects young adults, approximately 70% of whom are likely to be cured by modern first line combination chemotherapy (Canellos et al, 1992). Late side-effects of treatment, particularly gonadal toxicity and the risk of secondary myelodysplasia and acute myeloid leukaemia (MDS/AML), are major issues for long-term survivors.

In view of the high response and cure rates, randomized trials in this disease must recruit large numbers of patients to have adequate statistical power. Attempts to improve response and survival rates by intensifying initial chemotherapy must be balanced against the potential for long-term toxicity. In view of the effectiveness of salvage therapy, particularly high-dose therapy and autologous stem cell transplantation (ASCT) (Linch et al, 1993; Sweetenham et al, 1997; Yuen et al, 1997; Lazarus et al, 1999) intensified initial therapy may only be appropriate for patients with adverse prognostic factors at presentation.

The stakes are high for a young adult with advanced Hodgkin's disease. When asked to participate in a clinical trial, the patient must be reassured that this trial is asking a relevant question, that his or her chances of long-term survival will not be compromised by participating in the trial, and that the potential long-term consequences of the treatment will be addressed. The results of the British National Lymphoma Investigation (BNLI)/Central Lymphoma Group (CLG) randomized trial reported in this issue of the *British Journal of Cancer* (Hancock et al, 2001) should be judged against these criteria. This study was initiated in 1992, at about the same time that Cancer and Leukemia Group B (CALGB) reported the results of a randomized trial comparing ABVD (doxorubicin, bleomycin, vinblastine, dacarbazine), MOPP (mustine, vincristine, procarbazine, prednisone), and alternating MOPP/ABVD in 361 patients (Canellos et al, 1992). Response, failure-free and overall-survival rates were equivalent for ABVD and MOPP/ABVD. Both were superior to MOPP alone. Since the publication of this study, ABVD has been widely regarded as 'standard' chemotherapy for advanced Hodgkin's disease.

The BNLI/CLG trial compared an alternating 'variant' of MOPP/ABVD, known as ChlVPP/PABIOE (chlorambucil, vinblastine, procarbazine, prednisone/prednisone, doxorubicin, bleomycin, vincristine, etoposide) with PABIOE alone. The original phase II multicentre trial of the ChlVPP/PABIOE regimen plus radiotherapy reported 5 year FFS and OS rates of 68% and 78% respectively (Cullen et al, 1994). These results are comparable to MOPP/ABVD.

The authors' rationale for a randomized comparison of this regimen with PABIOE alone was 2-fold. Firstly, the CALGB study was relatively small and lacked sufficient power to confirm ABVD as the optimal regimen. Therefore, they felt a further comparison of an alternating regimen with an 'ABVD-like' regimen in a much larger trial was needed. Secondly, they justify the use of PABIOE alone on the basis of its 'similarity' to ABVD. However, as Table 1 shows, although 2 drugs are common to both regimens, they differ markedly, not only in the constituent drugs, but also in dose and scheduling. The assumption that the 2 regimens are similar was therefore speculative and apparently not supported by any phase II data demonstrating the efficacy of PABIOE alone. In the absence of such data, it is not clear why the authors felt that this regimen merited comparison in a large randomized trial, particularly since the resulting trial had no recognized standard arm. If ABVD had been chosen as the control arm of this trial, the ChlVPP/PABIOE regimen would have been assessed against an established standard, and the results of the study would have been informative. The choice of PABIOE resulted in a trial with little relevance to current therapy of Hodgkin's disease. This is particularly disappointing in view of the large number of patients (almost 700) who participated.

The overall survival (OS) for patients on both arms of this study (91% at 3 years for ChlVPP/PABIOE and 85% at 3 years for PABIOE) is comparable with many large clinical trials in advanced Hodgkin's disease. By contrast, the 3 year failure-free survival (FFS) rate of 58% for PABIOE is markedly inferior to that reported for ABVD in the CALGB trial. The poor outcome in comparison with ChlVPP/PABIOE resulted in early closure of the trial, although the interim analysis did not enable closure until accrual was only 21 patients short of the planned total of 700. The number of patients suffering from relapsed/progressive disease was almost double for PABIOE compared with the alternating regimen, and the number of patients treated with high-dose therapy and ASCT as second-line therapy was also double on this arm. Despite the apparent similarity of the 'salvage' strategy for both arms of the trial, the OS for those initially randomized to PABIOE is significantly lower than for the alternating regimen. This suggests that PABIOE is not only inferior first-line chemotherapy, but that its use may compromise the effectiveness of second-line treatment. This is an important observation which might be clarified if more details of second-line therapy were available.

The long-term toxicity of therapy is not addressed in this report. Since the alternating arm of this trial is clearly superior, late toxicity has not been a major factor in determining the best regimen. However, all trials in this disease should adopt the model established by major trials groups such as the EORTC (European Organization for Research and Treatment of Cancer) Lymphoma Co-operative Group an the NCI Canada, in which collection of long-term toxicity data is now routine.

**Table 1**

| ABVD | PABIOE |
|---|---|
| Doxorubicin 25 mg/m$^2$ i.v. days 1 and 15 | Doxorubicin 40 mg/m$^2$ i.v. day 1 |
| Bleomycin 10 IU/m$^2$ i.v. days 1 and 15 | Bleomycin 10 IU/m$^2$ i.v. days 1 and 8 (1st 4 cycles) |
| Vinblastine 6 mg/m$^2$ i.v. days 1 and 15 | Vincristine 1.4 mg/m$^2$ i.v. days 1 and 8 |
| Dacarbazine 375 mg/m$^2$ i.v. days 1 and 15 | Etoposide 200 mg/m$^2$ p.o. daily for 3 days |
| Treatment interval = 28 days | Prednisolone 40 mg/m$^2$ p.o. daily × 10 days |
| | Treatment interval = 21 days |

The immediate successor to the BNLI/CLG trial is now in progress, comparing ChlVPP/PABIOE with ABVD and a hybrid regimen, ChlVPP/EVA (chlorambucil, vinblastine, procarbazine, prednisone/etoposide, vincristine, doxorubicin) (Radford et al, 1995). Although the comparison of ChlVPP/PABIOE with a recognized standard regimen is now in progress, the group may now be asking the right question at the wrong time. New approaches to the treatment of advanced Hodgkin's disease have developed since the CALGB trial was reported. The description of a predictive model, based on clinical prognostic factors, has provided an opportunity to construct a risk-stratified approach to clinical trials (Hasenclever and Diehl, 1998). This is particularly important in view of the introduction of novel, dose-intensive regimens. These include the Stanford V (Bartlett et al, 1995) and dose-escalated BEACOPP regimens (Diehl et al, 1998), both of which are associated with significant short-term toxicity, but high-response and progression-free survival rates in phase II studies. The potential for long-term toxicity with Stanford V is thought to be low – early results show high rates of normal male and female reproductive function after completion of this therapy (Horning et al, 1998), and the risk for secondary MDS/AML is probably low. Toxicity with escalated BEACOPP is more worrying – in the latest report from the German Hodgkin's Lymphoma Study Group 8 of 460 patients randomized to this regimen have developed secondary AML/MDS (Diehl et al, 2000). However, the 3 year FFS for BEACOPP was 89%, compared with only 70% for the COPP/ABVD alternating regimen used in this trial. Most recent results for the Stanford V regimen are very similar, with a 5-year actuarial FFS of 89%.

The results of these regimens are exciting, and require confirmation in randomized trials, compared with standard therapy. A small randomized comparison of Stanford V with ABVD has recently been completed, although results are not yet available. A much larger comparison of these 2 regimens for 'low-risk' patients with advanced Hodgkin's disease is being performed in an inter-group study in the USA. Comparison of BEACOPP with ABVD will also be required to establish its true effectiveness. The assessment of these regimens is likely to be a major emphasis of clinical research in Hodgkin's disease for the next few years.

International collaboration will be required to perform clinical trials of sufficient statistical power to determine whether these new regimens represent true advances in treatment of Hodgkin's disease. Endpoints of these trials must include short- and long-term toxicity, as well as failure-free and overall survival. Patients with Hodgkin's disease can then be reassured that they are participating in trials which will answer relevant questions in a timely manner.

### REFERENCES

Bartlett NL, Rosenberg SA, Hoppe RT et al (1995) Brief chemotherapy, Stanford V, and adjuvant radiotherapy for bulky or advanced-stage Hodgkin's disease: a preliminary report. *J Clin Oncol* **13**: 1080–1088

Canellos GP, Anderson JR, Propert KJ et al (1992) Chemotherapy of advanced Hodgkin's disease with MOPP, ABVD or MOPP alternating with ABVD. *New Engl J Med* **327**: 1478–1484

Cullen MH, Stuart NS, Woodroffe C et al (1994) ChIVPP/PABIOE and radiotherapy in advanced Hodgkin's disease. The Central Lymphoma Group. *J Clin Oncol* **12**: 779–787

Diehl V, Franklin J, Hasenclever D et al (1998) BEACOPP, a new dose-escalated and accelerated regimen, is at least as effective as COPP/ABVD in patients with advanced stage Hodgkin's lymphoma: interim report from a trial of the German Hodgkin's Lymphoma Study Group. *J Clin Oncol* **16**: 3810–3821

Diehl V, Franklin J, Sieber M et al (2000) Dose escalated BEACOPP chemotherapy improves failure free survival in advanced Hodgkin's disease: updated results of the German Hodgkin's Lymphoma Study Group. *Blood* **96**: 576a

Hancock BW, Gregory WM, Cullen MH et al (2001) ChlVPP alternating with PABIOE is superior to PABIOE alone in the initial treatment of advanced Hodgkin's disease: Results of a British National Lymphoma Investigation/Central Lymphoma Group randomised controlled trial. *British Journal of Cancer*

Hasenclever D and Diehl V (1998) A prognostic score for advanced Hodgkin's disease. *New Engl J Med* **339**: 1506–1514

Horning SJ, Hoppe RT, Breslin S et al (1998) Brief chemotherapy (CT) (Stanford V) and involved field radiotherapy (RT) are highly effective for advanced Hodgkin disease's (HD). *Proc Am Soc Clin Oncol* **17**: 16a

Lazarus HM, Rowlings PA, Zhang M-J et al (1999) Autotransplants for Hodgkin's disease in patients never achieving remission: A report from the Autologous Blood and Marrow Transplant Registry. *J Clin Oncol* **17**: 534–545

Linch DC, Winfield D, Goldstone AH et al (1993) Dose intensification with autologous bone-marrow transplantation in relapsed and resistant Hodgkin's disease: results of a BNLI randomised trial. *Lancet* **341**: 1051–1054

Radford JA, Crowther D, Rohatiner AZS et al (1995) Results of a randomised trial comparing MVPP chemotherapy with a hybrid regimen, ChlVPP/EVA, in the initial treatment of Hodgkin's disease. *J Clin Oncol* **13**: 2379–2385

Sweetenham JW, Proctor SJ, Blaise D et al (1997) High-dose therapy and autologous stem cell rescue for patients with Hodgkin's disease in first relapse after chemotherapy: results from the EBMT. *Bone Marrow Transplantation* **20**: 745–752

Yuen AR, Rosenberg SA, Hoppe RT et al (1997) Comparison between conventional salvage therapy and high-dose therapy with autografting for recurrent or refractory Hodgkin's disease. *Blood* **89**: 814–822

© 2001 Cancer Research Campaign

